# Malarial Fever in the Sacramento Valley

**Published:** 1892-06

**Authors:** James T. Martin

**Affiliations:** Woodland, Cal.


### MALARIAL FEVER IN THE SACRAMENTO VALLEY.

JAMES T. MARTIN, M. D., WOODLAND, CAL.

Possibly you expect from me a definition of malaria before entering upon a brochure on this subject. But I have no such intention. I do not care to be caught in such a trap. The old word is good enough for me. At any rate, should another name be chosen you would still have to treat it in the same old way—that is, by the totality of the symptoms.

Prof. H. C. Allen, my old teacher, calls it "Marsh Miasm," but it comes stalking in with the same periodical regularity, bringing with it the same lank, lean, cadaverous form, smelling of the same stagnant pools, decaying vegetation and newly-turned-up soil as did his ancester, old malaria. It inhabits the same countries, believes in shaking up the same people, in fact, seems to grow in the richest and most productive soil. He will enter any class of society—rich or poor, good or bad—all alike may feel his blighting influence. In the chamber of the new-born babe you may see his gaunt and ghostly visage grinning ghastly at the mother long before she has fully recovered from the exhaustion of bringing another life into this uncharitable world. So it makes no difference by what name you call him, he presents the same old face, the same old aches, the same delirium, fever and sweat, howsoever disguised, as did his old ancestor whom you knew so well. Let it not be said that we always lift the same old club over his ungainly head, but rather that we select the one most suitable according to the *only* "law of selection" that has served so well on so many occasions. On three sides of us we have a system of irrigation ditches spreading wider and wider till most of the arable land in the vicinity can be flooded at any time of the year that the husbandman may desire. In addition to this, the outlet of Clear Lake, a small creek, from which the water is taken, flows about three miles north of the town, and sinks in the marsh near by. As a rule, during a portion of the growing season of the year, these ditches are entirely unused, hence, as a matter of course, in a fertile country, a luxuriant growth of vegetation springs up in and along these banks. This vegetation must necessarily die whenever the water is turned on for any length of time, so that when the gates are shut down and the water again dries out of the ditches, it leaves a great mass of decaying vegetation all along the beds of these artificial water-courses, exposed to the withering rays of the summer sun. Great quantities of this putrefying matter, when it becomes dry, rise into the air as an impalpable powder, and it is wafted by varying currents of air to different localities, seeking whom it may devour. Those living in the immediate vicinity of these ditches breathe a great deal of this atmosphere thus contaminated, and as a consequence are more subject to attacks of malarial fevers than those who live at a distance. The danger of an attack lessens as the distance from the ditches increases.

Then, again, to the east of us, lies an immense acreage of swamp land covered with a profuse growth of rushes known as "tules" that borders on the Sacramento River, and is from five to ten miles wide, partaking of the general course of the river. These lands are entirely submerged during a rise in the river sufficient to cause a break in the levee. This usually occurs in winters when we have an unusual amount of rain. The same levee that serves to keep the river in its banks also serves as a hindrance to the receding of the water, once the dikes have broken. Hence it is by a slow draining and evaporating process that the water is finally out of the tules. This usually occurs in July or August. It is therefore in the hottest season of the year that we are most exposed to the effects of this great mass of decaying vegetation from all sides. While the cold and damp continues, we do not feel the malarious influences nearly so much as we do when the sun shines the hottest.

In June or July every few years the great Columbia River overflows its banks, and it is often said that when the river again begins to return to its banks the people begin to shake. This is true, too, I believe, of all great river basins that are subject to overflow at or about this time of the year. Those who are studying the microbes of malaria find them in the greatest abundance in marshy regions, in stagnant pools, and, in fact, wherever there is plenty of rotting vegetable matter exposed to the direct rays of the summer sun.

The Board of Health, of New York city, advise sprinkling the streets as a means of protecting the inhabitants against any disease germs that would otherwise rise with the dust and be breathed by any one who came in contact with it. This, of course, is recommended as a sanitary measure, which we think well worth the attention of physicians generally.

There are three varieties of malarial fever that frequently occur in our section of the country which are worthy of attention. They might all, except one, however, be placed in the one class of masked intermittents. For our present purpose it will be sufficient to give you a typical case from each by way of illustration. In October, 1888, we were called into Capay Valley to see a young farmer living on the banks of the creek, who was, at the time, complaining very much of an intense headache, principally in the occipital region, but spreading over the head and finding another place of aggravation over the right orbit, very much worse on motion, especially sitting up. There was some photophobia with slight congestion of the conjunctiva. Temperature, 103 degrees; pulse, 120; perspiration, *very profuse*, indeed it was so excessive that his clothing and the bed-clothes were thoroughly soaked. This was during the hottest portion of the month, when the thermometer stood at 100 degrees F. in the shade. Yet this man had, besides his working clothes, most all the available bed-clothes in the house piled on to keep him warm. He complained of heat, yet was afraid to uncover lest he should become cold. There was very much aching in different parts of the body, which was especially severe in the lumbar region, with marked tenderness in the region of the liver and spleen. On making inquiry about the case we found that after two or three days of malaise, fever came on the day before our visit, and still continued to rise till we saw the patient on the afternoon of the 8th of October. Some time during the night the fever subsided, and on the 9th apyrexia appeared, which lasted about twenty-four hours, when the temperature began again to rise and continued to do so for nearly forty-eight hours, and then dropped to normal again, only to repeat the same process during the three days following. This is about the course of all this class of fevers. Two days of increasing fever, that falls rapidly to normal on the third day, with the exception that in the more serious cases the temperature often drops down below normal—in one case noted as low as 96½ degrees. The maximum rise in temperature in these more severe cases is in the neighborhood of 105 or 106 degrees. Persons who are subject to attacks of this fever live either on an irrigation ditch, the creek, or on some other water-course. The progress of cure is worthy of note. After the first correct prescription the fever begins to rise later and later in the day with each recurring paroxysm until no fever appears until the morning of the second day, when it is ushered in by a slight chill. One would naturally think that we would have a paroxysm every third day, but we don't, for about this time it changes to an every-other-day intermittent. Now, as the fever comes later and later in the day, the chill becomes harder and harder, till we have a very hard paroxysm between ten and eleven o'clock in the forenoon and lasting from one to two hours. From this time on the disease usually follows about the course of an old-fashioned intermittent fever. We have gotten the mask off which never came off when Quinine was given; it either afforded a temporary relief, with much aggravation of all the symptoms when they did return, or else changed its character entirely into a lingering typho-malarial or typhoid fever that is so common, and has caused so much trouble in malarial districts. We know of no other drug that is so often misused as Quinine in malarial districts. As long as the name "malaria" lives, there will always be some excuse hatched up for the use of this drug, no matter how much a patient may protest that Quinine is injurious to him, makes him deaf or hurts his stomach, yet he gets it just the same, disguised in some of the thousand and one ways that are so convenient when it is desirable to keep the patient in darkness as to what he has swallowed. How an intelligent physician can give a medicine year in and year out without learning that it often does injury, is more than I can understand. It does seem to me that it is one of the special prerogatives of the physician to know the difference between drug and disease symptoms, and therefore know when disease symptoms end and drug symptoms begin or become manifest. The class of fevers herein above mentioned are very prone to return, usually at about the same time the next year. Though this is not constant, some return the following spring or fall as the case may be. But whenever they return it is always as intermittents. Aside from the causes already given for malarial fever in general, this particular variety has for its immediate and exciting cause, living on an irrigating ditch, working in the hot sun, and drinking large quantities of water. There may be other causes, but these are the most prominent.

The next and simplest of all forms of malaria shows fever in the morning, continuing through the day, declining sometime in the night, only to rise again next morning and repeat the same process. There is very seldom any headache, perspiration slight, if any. The patient complains of a tired, weak feeling, with a loss of appetite, complexion sallow, bowels constipated, temperature seldom or never rises higher than 102 degrees; they are not very sick, but often think they are going to be. Boys and young people—especially those who have been in the habit of bathing in some of the various water-ways in the country—are more liable to this fever than others. In fact, most all the cases we have had were directly attributable to this cause. The tongue is heavily coated, usually a dirty gray. Taste often spoken of as *very bad*, and quite often nothing tastes good except water. This disease runs somewhere from three to ten days, owing perhaps to the amount of exposure before the attack is precipitated.

The recovery is usually clear without either bad results or relapses.

In the next variety the mask is thrown off entirely, and we have the bold, angular type of the old-fashioned intermittent with its chills, fever, and sweat in regular paroxysms. It is not necessary for us to describe it here; you all know him and are able to recognize him anywhere. If not, your patient will introduce you to him.

As I have before intimated, the cause of all these fevers is stagnant water, decaying vegetation, and a hot sun. In recent investigations scientists have declared that they have discovered the microbe which is concerned in the propagation of malarial fever. Of course, the aforesaid malarial country must exist where the microbe can propagate, or he won't exist to work out his pet theories on an unoffending populace; no matter how many times he may shake up the body that he has seen fit to attack, or how many times he may multiply in that body, he is perfectly impotent of harm when through with that body, so far as other persons are concerned. The suggestion therefore comes to me, why not disinfect the country, work off your germicides on the stagnant pools and decaying vegetation, and dispose of Mr. Microbe before he is sufficiently developed to attack the unwary. Now, I am not much acquainted with the most fashionable germicides and microbe-killers of the day, but somehow it seems to me that it will take pretty nearly as great a quantity of any one of them to kill *that* much-talked-of infusoria as it would to kill a man. There is one thing, however, that I do know, the well-directed similar remedy will make the human body so healthy that Mr. Micrococcus finds it exceedingly uncomfortable to remain, and hence moves out. Healthy blood, we are told, will actually kill 46,000 of these little animalculæ in a moment of time. Therefore as your remedy brings health to your patient Mr. Microbe and all his relatives must take immediate and unceremonious departure, or meet their doom in the onward flow of that life-giving fluid made pure in obedience to law. The elements which enter into the cure are somewhat varied. The very profuse perspiration that is noticeable flushes the pores and carries away an immense amount of *débris* that has been collecting for a long time, damming up these little sewers of the body and causing the overflow which poisons the system. Frequent bathing with good, pure water will be found not only very grateful to the patient, but very beneficial as well, and is one of the best adjuvants to the treatment. There are very few, if any, other local measures that are of any avail at all. It goes without saying that the medicinal treatment is the indicated remedy.

Since writing the above, a new theory of intermittent fever has been published by Dr. Mathew D. O'Connell in the *Indian Medical Gazette* for November, 1891. While not questioning the presence of micro-organism, either animal or vegetable, in the blood, the Doctor thinks that another hypothesis is not only possible, but fully adequate to explain the facts of ague and its consequences. This hypothesis as quoted by the *Medical Record* of January 30th, 1892, says that ague or intermittent fever is due to an intermittent excess of water in the blood, and that this intermittent excess of water in the blood, or hydræmia, is the obvious result of exposure to meteorological surroundings (heat and moisture) which go to make up what we call a malarious climate. The facts which have led him to the belief that intermittent fever is due to an intermittent hydræmia are as follow:

1. There is known to be excess of water in the blood in all fevers.

2. Excess of water in the blood, or hydræmia, will cause fever.

3. The meteorological conditions (heat and moisture) which make up what we call a malarial climate are such as must, by preventing its excretion, produce an intermittent excess of water in the blood, or hydræmia.

4. Free elimination of water from the blood of a person suffering from ague reduces the temperature to normal.

5. Excess of water in the blood, especially if intermittent, will produce all the usual sequelæ of ague, viz.: (a) enlargement of spleen; (b) leucocythæmia; (c) pigmental deposits in organs.

6. All successful treatment of ague reduces the quantity of water in the blood.

7. Change of climate cures the disease.

I am neither a prophet or a son of a prophet, but I believe that somewhere in the varying cycles of time we will have this theory or one very much like it as the accepted pathology of malarial fevers. So the changes come, the old pathology gives place to the new, our knowledge increases, and we are brought out of the slough of despond into a clear and running stream with new and refreshing surroundings only to float down again into the same old swamp.

